# Protective or Harmful: The Dual Roles of Autophagy in Diabetic Retinopathy

**DOI:** 10.3389/fmed.2021.644121

**Published:** 2021-03-25

**Authors:** Qiaoyun Gong, Haiyan Wang, Ping Yu, Tianwei Qian, Xun Xu

**Affiliations:** ^1^Shanghai Key Laboratory of Ocular Fundus Diseases, Department of Ophthalmology, National Clinical Research Center for Eye Diseases, Shanghai Engineering Center for Visual Science and Photomedicine, Shanghai Engineering Center for Precise Diagnosis and Treatment of Eye Diseases, Shanghai General Hospital, Shanghai, China; ^2^Department of Pharmacy, Ruijin Hospital, Shanghai Jiao Tong University School of Medicine, Shanghai, China

**Keywords:** autophagy, diabetic retinopathy, mitophagy, AMPK pathway, mTOR

## Abstract

Autophagy is a self-degradative pathway involving intracellular substance degradation and recycling. Recently, this process has attracted a great deal of attention for its fundamental effect on physiological processes in cells, tissues, and the maintenance of organismal homeostasis. Dysregulation of autophagy occurs in some diseases, including immune disease, cancer, and neurodegenerative conditions. Diabetic retinopathy (DR), as a serious microvascular complication of diabetes, is the main cause of visual loss in working-age adults worldwide. The pathogenic mechanisms of DR are thought to be associated with accumulation of oxidative stress, retinal cell apoptosis, inflammatory response, endoplasmic reticulum (ER) stress, and nutrient starvation. These factors are closely related to the regulation of autophagy under pathological conditions. Increasing evidence has demonstrated the potential role of autophagy in the progression of DR through different pathways. However, to date this role is not understood, and whether the altered level of autophagy flux protects DR, or instead aggravates the progression, needs to be explored. In this review, we explore the alterations and functions of autophagy in different retinal cells and tissues under DR conditions, and explain the mechanisms involved in DR progression. We aim to provide a basis on which DR associated stress-modulated autophagy may be understood, and to suggest novel targets for future therapeutic intervention in DR.

## Introduction

The occurrence of diabetes mellitus (DM) globally is increasing markedly, and is estimated to reach 7.7% by 2030 (1). It is well-recognized that many complications result from uncontrolled diabetes, affecting the cardiovascular system and contributing to kidney failure, peripheral vascular diseases, and other pathologies (2). Diabetic retinopathy (DR) is a microvascular disease resulting from DM and is the leading cause of visual dysfunction and blindness in adults worldwide (3). DR is initially induced by long periods of extremely high glucose (HG), and is characterized by breakdown of the blood–retinal barrier (BRB), with increased permeability and neovascularization (4). However, the detailed molecular and pathologic mechanisms of DR are not fully understood.

Autophagy is a catabolic process that involves the degradation and recycling of cellular components via a lysosomal mechanism. In response to intra- and extracellular stresses, autophagy acts as a sensor to induce the degradation of cellular constituents, which are re-used to provide the nutrients required for cellular homeostasis (5). The process of autophagy can be classified into three main types, including macroautophagy (the widely recognized form of autophagy), chaperone-mediated autophagy (CMA), and microautophagy. These types are differentiated according to the process of degradation. Autophagy occurs in all kinds of cells and tissues, and a normal baseline of autophagy is maintained to remove damaged cell constituents (6, 7).

Autophagy has been found to play a role in DM (8), and is modulated and involved in DR. Increasing evidence has shown that factors related to DR including hyperglycemia, oxidative stress, hypoxia, endoplasmic reticulum (ER) stress, and nutrient starvation are associated closely with the activation of autophagic flux (9). In this review, we aim to clarify whether modulations in the autophagic process are protective or harmful, and how autophagy influences the development of DR. The recognition of molecular and pathogenic mechanisms underlying this autophagic process may be useful in the development of strategies for DR prevention or treatment.

## Diabetic Retinopathy

In the past 20 years, the prevalence of DM has risen and DR, the most common vision-threatening complication of DM, has attracted a great deal of attention. DR results from abnormal retinal blood vasculature and may be non-proliferative (non-proliferative DR, NPDR) or proliferative (proliferative DR, PDR). The retinal microvascular circulation is negatively influenced by diabetes, which causes a series of structural abnormalities. In the early phase of DR, tissues and cells are in a chronic state of hyperglycemia, causing damage to the retinal microvasculature, including pericyte apoptosis, tight junction impairment accompanied by increased vascular permeability, and capillary occlusion (10–12). These changes occur in a non-proliferative period of DR and simultaneously result in BRB breakdown, altered proliferation of endothelial cells, edema. and retinal microvascular degeneration (13). As DR develops, these non-proliferative changes are influenced by retinal ischemia and hypoxia, resulting in increased expression of various vascular growth factors and mediators of inflammation, oxidative stress, ER stress, and accumulation of advanced glycation end products (AGEs) (13–15). These alterations induce retinal neovascularization and the formation of fibrovascular membranes, which are features of the proliferative stage of DR, and may cause vitreous hemorrhage and retinal detachment. Moreover, visual impairment caused by edema affects the macula at the center of the retina (termed diabetic macular edema, DME). DR is the main cause of visual disability in patients with DM (4) and an understanding of the molecular mechanisms involved is important as a basis for more effective and specific targets for prevention and treatment.

A crucial characteristic of DR is breakdown of the BRB, which may occur at any phase of this disease, taking place from edema and exudation (a NPDR phase) to highly permeable neovascularization (a PDR phase) (16). The BRB, which consists of the retinal vasculature (inner) and the retinal pigment epithelium (RPE, outer), eliminates cytotoxic substances from circulating inflammatory cells and the neural retina, thus helping to maintain the extracellular chemical composition of the retina (17). Endothelial cells (ECs) of retinal capillaries form the inner BRB, with tight junctions almost impermeable to protein exchange (16). The outer BRB is localized at the RPE between the choroid and retinal photoreceptors. RPE cells help maintain retinal fluid balance (18) and photoreceptor function (19). Photoreceptor outer segments (OS), where the pigment rhodopsin absorbs incident light, are extended constantly by the addition of cell membrane disks. The oldest membrane disks are located in the sub-retinal space, where they are digested by RPE cells via a process known as phagocytosis, which involves recycling of the component molecules back to the retina to form new OS (20). In addition, neural and glial cells release metabolic products such as nitric oxide (NO) and lactate, optimizing retinal blood flow according to metabolic needs. Changes in the interactions between vascular and neural cells contribute to vascular dysfunction in the pathogenesis of DR (3).

**Figure 1:**
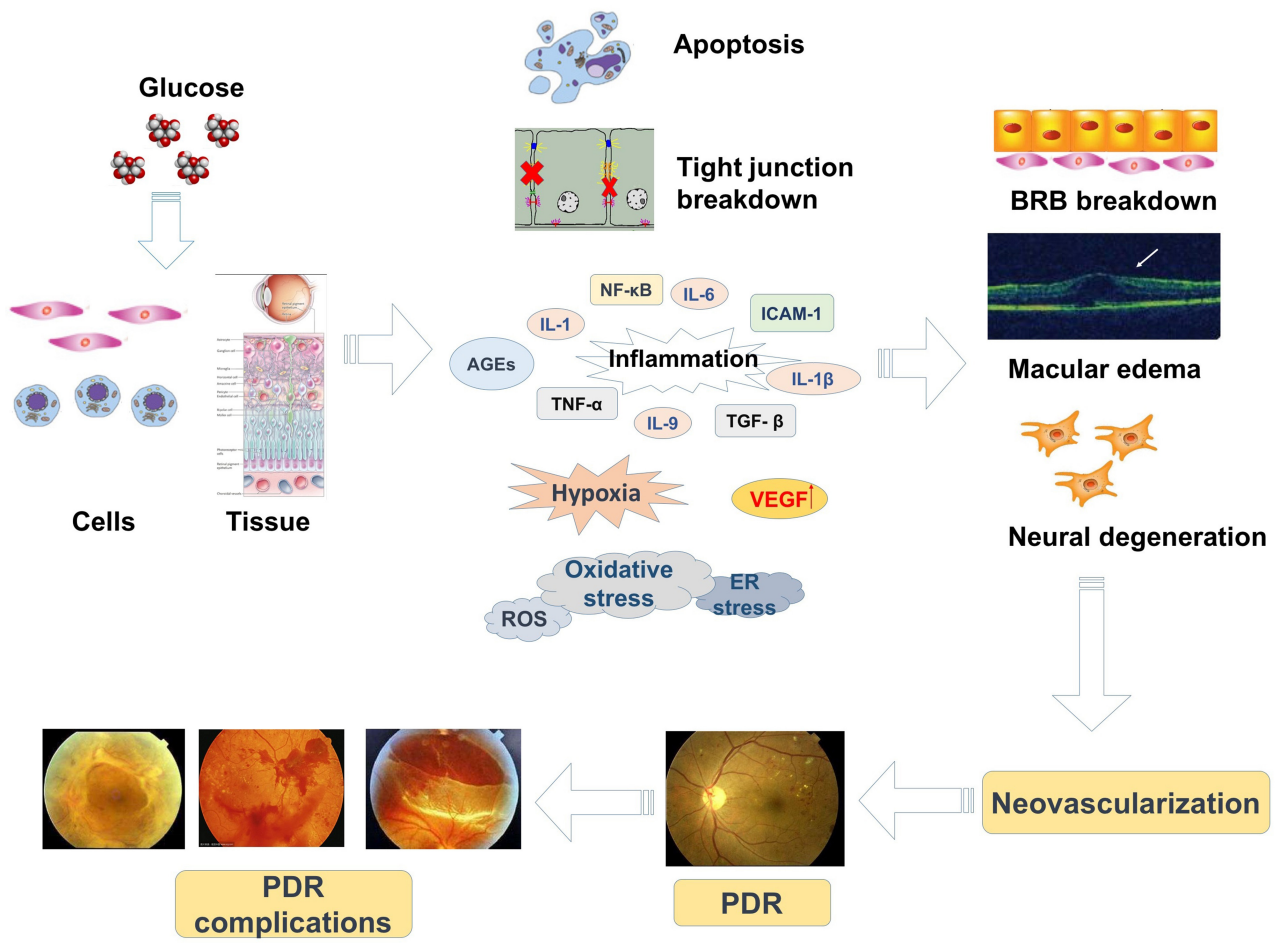
The pathogenic mechanisms and progression of DR.

Hyperglycemia is the initiating causative element in the pathogenesis of DR and induces a series of reactions including modified cellular signal transduction. Impairment of ECs and pericytes causes an imbalance in the modulation of blood flow with subsequent ischemia, hypoxia, oxidative stress, and retinal edema (3, 12). The oxidative damage in diabetic tissues is due to increased expression of reactive oxygen species (ROS) or a decreased ability of the retina to manage oxidative stress (8). In DR, it is thought that mitochondrial dysfunction is induced by oxidative damage (21). Mitochondrial DNA is initially protected by short-term compensatory mechanisms which become overwhelmed by persistently high glucose (22). In diabetic retinas, AGEs can modify proteins that increase oxidative stress, upregulate inflammatory cytokines, and change vascular function (23). The evidence suggests that both oxidative stress and chronic inflammation contribute to the progression of DR. Moreover, the induction of oxidative stress promotes mitochondria to produce superoxide in ECs, arouses inflammatory mediators, and triggers angiogenesis (24). Alternatively, the enhanced expression of CCAAT/enhancer-binding protein (C/EBP), homologous protein growth arrest, and DNA damage-inducible gene 153 (CHOP/GADD153) in retinas of diabetic rats and in human retinal capillary ECs exposed to high glucose may assist the initial progression of DR via ER stress (25). Therefore, mechanisms related to hypoxia, oxidative stress, inflammation, ER stress, and microvascular dysfunction are involved in the pathological progression of DR (Figure 1).

## Autophagy

### Forms of Autophagy

Autophagy involves the degradation and re-utilizing of cytosolic constituents under physiological or pathologic conditions and is a self-degradative pathway. It plays crucial roles in physiological processes among cells, tissues and the maintenance of organismal homeostasis (26). As outlined earlier, three forms of autophagy occur in mammalian cells including macroautophagy, CMA, and microautophagy. While each involves a different process, all culminate in delivery of constituents to lysosomes for degradation and reuse. To capture the substance from the lysosomes, macrophagy involves synthesis of double-membrane vesicles (autophagosomes) and their transportation to the lysosome (27). Macroautophagy, well-known as “autophagy,” is a conserved lysosomal degradation pathway. Autophagy maintains cellular quality by mediating the degradation of protein aggregates, lipid droplets, damaged organelles, and intracellular pathogens, and maintains cellular bioenergetics by recycling cytoplasmic components (28). CMA uses chaperones to identify and sequester cargo proteins with a specific pentapeptide motif. The substrates are then flattened and translocated individually directly across the lysosomal membrane (29). CMA takes place only in mammalian cells and is thought to be responsible for the elimination of over 30% of intracellular proteins (30). The proteins include a KFERQ-like motif in their amino acid sequence, which is recognized by the constitutive cytosolic chaperone Hsc70 (heat shock cognate of the Hsp70 family), leading to their delivery to the lysosome. During the process of microautophagy, invaginations or protrusions of the limiting lysosomal membranes occur to trap cargo, and can envelope intact organelles (31). Thus, material intended for degradation arrives at the lysosomal lumen and is engulfed.

### The Regulation of Autophagy

Autophagy is induced by intra- and extracellular stress, including nutrient starvation, oxidative stress, ER stress, hypoxia, and pathogen infection. It is recognized that the autophagic process is closely associated with the AMPK (adenosine monophosphate-activated protein kinase) and mTOR (mammalian target of rapamycin) mediated signaling pathways, which are the two prime pathways accountable for regulating the nutritional condition of cells (32). With the detection of autophagy-related genes (ATG) in yeast genetics and mammalian homologs (33), the essential machinery of autophagy has been studied and classified. In yeast, ATG1 was initially recognized from genetic screens, a serine-threonine kinase, and linked with autophagy in cellular metabolism. Like other ATG proteins, ATG1 also has its mammalian ortholog. ATG1 possesses high homology with C. *elegans* uncoordinated-51 (UNC-51), which contains two mammalian homologs acknowledged as UNC-51 like autophagy activating kinase 1 (ULK1) and ULK2 (34). ATG1/ULK1 complex, usually recognized as an initiator in the autophagic process, plays important roles in activation of an autophagic response both in mammalian and yeast cells (32). mTOR may have two direct signaling complexes, as mTOR complex 1 (mTORC1) and mTORC2, by combining with companion proteins (35, 36). A recognized upstream modulator of mTORC1 is the PI3K/AKT signaling pathway and the growth factor (37, 38). For its role in nutrition sensing, mTORC1 is activated by an abundance of amino acids (39). AMPK is known as a sensor of cellular energy levels and is stimulated by a high AMP/ATP ratio. AMPK can directly phosphorylate RAPTOR, hence leading to inhibited mTORC1 activity through allosteric abolition (40). It has also been shown that the increased cytosolic Ca^2+^ concentrations due to ER stress result in calcium/calmodulin-dependent protein kinase kinase 2, beta (CAMKK2/CaMKKb) to induce AMPK, and inhibit mTOR (41). Therefore, a crucial role of AMPK is related to mTORC1 modulation in linking cellular energy level and autophagy.

Inhibition of mTORC1-induced autophagy by genetic or pharmacologic means and the mechanisms involved have been investigated and reported. In yeast, a nutrient-rich condition, ATG13 is activated and phosphorylated partly through the nutrient-sensing kinase TOR, which is thought to inhibit its connection with ATG1 (42, 43). Considering this model, upon nutrient starvation, TOR is inhibited, resulting in ATG13 dephosphorylation and subsequent disruption of ATG1-ATG13 interaction and autophagy. Thus, the modulated ATG1-ATG13 interaction monitors the autophagic function of ATG1 and the ATG1 complex. In mammalian cells, the main autophagic ULK1 complex consists of ULK1, ATG13, and RB1CC1 and regulates the signal of the nutrient-sensing kinase mTOR (44, 45). mTORC1 can prevent the autophagy-initiating ULK complex by phosphorylating ATG13 and ULK1/2 (45). Moreover, mTORC1 can phosphorylate Ser758 (Ser757 in mouse) of ULK1, inhibiting the phosphorylation and interaction of ULK1 by AMPK, which is crucial for ULK1 activation (46). Moreover, AMPK and mTORC1 also mediate the regulation of VPS34 complex, a class III PI3K whose activity is fundamental for autophagosome formation. VPS34 complexes play essential roles in cellular vesicle tracking and autophagy stimulation. In particular, the ATG14L-associated VPS34 complex is involved in autophagy modulation. In conditions of stress, AMPK induces the proautophagy VPS34 complex through phosphorylating Beclin 1, whereas it then prohibits the non-autophagy VPS34 complex by phosphorylating Thr163/Ser165 in VPS34 (47). Conversely, mTORC1 phosphorylates ATG14L in the VPS34 complex and simultaneously prevents the lipid kinase activity of VPS34, suggesting another mTORC1-associated mechanism in inhibiting autophagy (48).

Although autophagy is mainly modulated by protein levels, long-term stress can also enhance transcriptional expression of autophagy genes, one of which is the transcription factor EB (TFEB) (49). The transcriptional level of TFEB is modulated by nutrient shift and phosphorylation-dependent cytoplasm-to-nucleus shuttling (50). It has also been reported that mTORC1 can directly phosphorylate TFEB at sites of Ser142 and Ser211, and the phosphorylation activity leads to cytoplasmic sequestration of TFEB (51). In summary, mTORC1 tightly suppresses autophagy flux through phosphorylation-dependent abolition of ATG1 complex, ULK1/2, and the VPS34 complex, inhibiting the expression of lysosomal and autophagy genes through transcriptional regulation of TFEB phosphorylation.

In addition, damage-regulated autophagy modulator (DRAM) is a lysosomal protein that induces macroautophagy and contributes to apoptosis in a p53-independent manner (52, 53). DRAM is expressed and highly conserved among kinds of species including humans, mice, and et al. DRAM localizes in lysosomes or endosomes, which plays an essential role in the formation of autophagic lysosomes. Overexpression of DRAM promoted the punctate distribution of the autophagy marker LC3 in the cytoplasm (54). By analyzing the genomic sequence, DRAM is a target gene of p53. Therefore, autophagy induced by p53 accumulation is mediated by DRAM. Meanwhile, DRAM is not only crucial for autophagy aroused by p53 overexpression or DNA damage, but also indispensable for p53-caused apoptosis (55). Although DRAM alone has a weak potential to trigger apoptosis, if DRAM is lacked in DNA damage or other apoptotic stimuli, p53-caused apoptosis will be severely depressed, revealing that p53-induced apoptosis relies on the presence of DRAM (52). Therefore, DRAM is not only highly associated with the stress-caused regulator of autophagy but also adjusted the p53 mediated damage-induced programmed cell death.

## The Beneficial Effects of Autophagy on Diabetic Retinopathy

### Autophagy Plays Neuroprotective Roles in DR

The understanding of DR as a microvascular disease has advanced, and it is now recognized that neurodegeneration plays a vital role in this complication of DM (56). Alterations of autophagy may occur in multiple neurodegenerative pathologies (57). Retinal ganglion cells (RGCs) are projection neurons of the vertebrate retina and their loss is observed in various eye diseases. In the study of retinal hypoxic–ischemic events including DR, Russo et al. (58) studied the autophagic reaction in the retinas of mice treated with ischemia caused by transient increase in intraocular pressure, presenting a biphasic and reperfusion time-dependent regulation of the process. They found that the early enhancement of autophagy restricted RGC death, while the induction of autophagy was exhausted 24 h after the ischemic event. The reduced autophagic flux was accompanied by increased SQSTM-1/p62, inhibited ATG12-ATG5 conjugate, Beclin1, and ATG4 expression. Furthermore, during the reperfusion process, AMPK is inactive, whereas a continuous phosphorylation of Akt may be found and the transient overexpression of mTOR was found after 6 h of reperfusion. These findings revealed that the inhibition of AMPK and activation of Akt/mTOR regulated the decreased autophagy in the later phase of reperfusion. Animal fasting or subchronic systemic treatment with rapamycin retained and enhanced autophagy and then promoted RGC survival, suggesting that modification of autophagy may be a potential strategy in treating retinal neurodegenerative diseases related to hypoxic/ischemic stresses (58).

As neuronal impairment plays a crucial role in DR, Amato et al. proposed the hypothesis that the balance between neuronal survival and death may rely on a similar equilibrium between autophagy and apoptosis. Murine retinal explants were exposed to high glucose (HG, 75 mM) for 10 days. HG-treated explants showed a significant decrease in the autophagic flux paralleled by an increase in apoptosis, which was due to the enhanced expression of mTOR. Following treatment with octreotide (OCT, a neuroprotectant), HG-treated retinal explants recovered from apoptosis and autophagic flux was induced, accompanied by mTOR inhibition. Following inhibition of the autophagic activity with chloroquine (an autophagy inhibitor), the anti-apoptotic effect of OCT was completely abolished. Immunohistochemical experiments indicate that OCT-induced autophagy is localized at bipolar and amacrine cells, and the ganglion cell layer (GCL). The antithetic roles of autophagy and apoptosis suggest that their equilibrium is important for neuronal survival. Thus, autophagy plays a neuroprotective role in DR and may be an important target for treatment strategies (59).

Glaucoma and DM are the two prime causes of selective RGC death. Park et al. identified autophagy and the associated pathways in glaucomatous and diabetic retinas, and investigated their influence on RGC survival. Beclin-1 and LC3B-II/I were moderately enhanced in diabetic retinas, while they were significantly increased at 4 and 8 weeks after glaucoma induction. Increased phosphorylated AMPK and decreased phosphorylated mTOR were also observed in the diabetic retinas. Inhibition of autophagy by 3-MA increased RGC apoptosis in diabetic retinas, but inhibited their apoptosis in glaucomatous retinas (60). This suggested that RGC death is differentially modulated by autophagy depending on the triggering injury, and that in the diabetic retina, autophagy induced by AMPK activation may support survival of RGCs.

These studies confirmed that the activation of AMPK and the inhibition of mTOR induce autophagy flux and thus mediate RGC cell protective functions to ameliorate the neurodegeneration in DR.

### Mitophagy, a Specialized Form of Autophagy Works to Ameliorate DR Progression

To understand the biochemical pathways involved in the physiologic abnormalities of retinal cells in DR, several studies have focused on mitochondrial homeostasis and the signal transduction pathways involved in supporting mitochondria (61, 62). Mitochondria play a vital role in the modulation of autophagy, apoptosis, inflammation, and oxidative stress (62, 63). Impaired and dysfunctional mitochondria are found in the retinas of diabetic patients (64) and diabetic rodents (65) and their effective and selective removal is vital for sustaining mitochondrial homeostasis. Mitophagy, a specialized form of autophagy, is recognized as the core mechanism for selective degradation of mitochondria, controlling mitochondrial quality and quantity (66). Mitophagy operates via two mechanisms, autophagic recognition of mitochondria through a BNIP3L–LC3 interaction and the PINK1-Parkin pathway (67).

Zhou et al. (68) observed decreased retinal thickness, retinal vascular degeneration, and destroyed retinal function in db/db mice, and the amelioration of these changes by treatment with Notoginsenoside R1 (NGR1, 30 mg/kg) for 12 weeks. Pretreatment with NGR1 could decrease apoptosis, inhibit vascular endothelial growth factor (VEGF) expression, enhance pigment epithelium derived factor, and prohibit inflammation and oxidative stress *in vivo* in diabetic retinas from db/db mice and HG-treated rat retinal Müller cells (rMC-1) (60 mM). Moreover, the number of mitophagy autophagosomes was increased in diabetic retinas and even increased in NGR1-treated db/db mice. *In vitro* observation of rMC-1 cells using MitoTracker and GFP-LC3 puncta under hyperglycemia found that NGR1 increased mitophagy. NGR1 pre-treatment also improved expression levels of Parkin and PINK1, upregulated the LC3-II/LC3-I ratio, and suppressed the expression of p62 in rMC-1 cells exposed to HG in db/db mouse retinas. Thus, NGR1 may ameliorate DR through PINK1-dependent activation of mitophagy (68).

In research on mitochondrial quality control in neurodegenerative diseases, Hombrebueno et al. (69) showed that mitophagy is impaired in DR and is decoupled from mitochondrial biogenesis during DR development. In diabetic mitophagy-reporter mice (*mitoQC-Ins2*^*Akita*^), mitochondrial loss increased due to the failure of mitochondrial biogenesis to compensate for diabetes-exacerbated mitophagy. *In vivo* (retinas of hyperglycemic Ins2^Akita/+^ mouse) or *in vitro* [primary Müller cells and MIO-M1 cultures with HG (30.5 mM)] models of DR show enhanced mitophagy, as indicated by the increase of PINK1 gene, upregulated LC3B+ autophagosomes and reduced autophagy substrate p62/SQTSM. In prolonged diabetes, impairment of mitophagy is associated with the progression of retinal senescence, a phenotype which inhibited HG-induced mitophagy in *mitoQC* primary Müller cells. This study revealed that normalizing mitochondrial turnover may protect mitochondrial quality control and provide therapeutic options for the treatment of DR-associated complications (69).

Inhibition of mitophagy by HG has also been found in another retinal cell type, RPE. Zhang et al. (70) demonstrated that HG (50 mM) accelerated cell apoptosis and ROS generation while decreasing proliferative abilities as well as cell mitophagy. The study found significantly lower LC3B-II/I ratio in the HG group, p62 accumulation, and increases in pro-apoptotic proteins including Bax, cleaved Caspase 3, and cleaved Caspase 9. The detrimental effects of hyperglycemia on RPE are ameliorated by ROS scavengers and aggravated by autophagy inhibitor 3-MA or mitophagy inhibitor cyclosporin A (CsA). The HG-induced inhibition of cell proliferation, downregulation of mitophagy, and increased cell apoptosis were achieved by ROS mediated inactivation of the PINK1/Parkin signal pathway (70). Conversely, Huang et al. (71) found that ARPE-19 cells exposed to HG (30, 50, and 70 mM) stress demonstrated a significant decrease in LC3-I expression and clear increase in the number of autophagosomes, elevated intracellular ROS level, and enhanced expression of PINK1, Parkin, BNIP3L, and LC3-II. Pretreatment of ARPE-19 cells with *N*-acetyl cysteine (NAC) or 3-MA under hyperglycemic conditions led to a significant decrease in the expression of PINK1, BNIP3L, and LC3-II. Thus, autophagy may protect ARPE-19 cells against HG-induced injury by regulating the PINK1/Parkin pathway and BNIP3L to mediate mitophagy (71).

Mitophagy is recognized as a form of autophagy in which dysfunctional mitochondria are specifically targeted by lysosomes for autophagic degradation to inhibit proinflammatory activation. The degradation modulates inflammasomes and prohibits intracellular signaling by eliminating damaged mitochondria, which could otherwise induce elevated amounts of intracellular ROS (72). Shi et al. (73) treated ARPE-19 cells with HG stress (30 mM) and found that they respond with an enhancement in autophagy. In contrast, 3-MA restricted autophagy flux, caused the accumulation of damaged-mitochondria-producing-ROS, the upregulation of NLRP3 inflammasome, and subsequently induced IL-1β secretion (73). It is recognized that the generation of ROS leads to an increase in NLRP3 inflammasome via release of the ROS-sensitive NLRP3 ligand thioredoxin-interacting protein (TXNIP) from its inhibitor thioredoxin (TRX) (74). Thus, autophagy-induced cytoprotection was dependent on the NLRP3 inflammasome activation modulated by mitochondrial ROS.

### Autophagy Mitigates DR by Inhibiting Apoptosis

Autophagy, as a crucial and evolutionarily conserved mechanism to sustain cellular homeostasis, is tightly associated with the apoptosis regulated by common intracellular stress, leading to cross-talk in various diseases with both processes having mutual influence (75). For example, in response to ER stress, there are common upstream signaling pathways between autophagy and apoptosis, involving PERK/ATF4, ATF6, IRE1α, and Ca^2+^ (76–78). Autophagy can inhibit the activation of apoptosis by suppressing the stimulation of apoptosis-related caspase which could ameliorate cellular injury, while it also helps trigger apoptosis (79). Moreover, the upregulation of apoptosis-associated proteins can conversely depressing autophagy by inhibiting autophagy-related proteins, including Beclin-1, ATG3, ATG4D, and ATG5 (80). Although the interactions of different autophagy- and apoptosis-related proteins, and also common upstream signaling pathways have been found, the potential regulatory mechanisms have not been clearly understood. Especially, apoptotic pathways are thought to have a role in the death of retinal cells under detrimental conditions involving diabetic stress, while autophagy has also been found to participate in the fate of stressed retinal cells (57, 81). Therefore, the equilibrium between autophagy and apoptosis in retinal cells during different phases of DR deserves further attention.

In rat Müller cells, Wang et al. (82) found that HG (40 mM) induced a downregulation in autophagy and an upregulation in apoptosis. Epigallocatechin gallate (EGCG, a major polyphenol in green tea) enhanced autophagy by improving the formation of autophagosomes, elevating lysosomal acidification, and activating autophagic flux in Müller cells under HG. EGCG may improve cell viability by enhancing autophagy. In addition, EGCG inhibited the reactive gliosis of Müller cells and reduced retinal damage. These findings highlighted that HG decreased autophagy, caused P62 accumulation resulting from lysosomal dysfunction, and then induced apoptosis which could be reversed by treatment with EGCG. Furthermore, EGCG upregulated autophagic flux by modulating the mTOR-mediated pathway in HG-treated Müller cells. Thus, EGCG may increase capacity for Müller cell proliferation and inhibit apoptosis by inducing autophagy under HG conditions (82).

Consistent with Wang et al. (82), Lopes De Faria et al. (83) found that in cultured rMC-1, hyperglycemia (25 mM) induced the upregulation of early and late autophagic markers (LC3II/I, Beclin-1), accumulation of p62 as well as ER stress with apoptosis augmentation. Inhibition of autophagy under HG conditions caused an increased rMC apoptotic rate. By silencing p62, ER stress was decreased and apoptosis was prevented. On activating the autophagy in HG-treated rMC cells with rapamycin, the cargo degradation was re-established, VEGF increase was prevented, and apoptosis was reduced. This study suggested that HG enhanced autophagy but prevented p62 cargo degradation due to lysosomal dysfunction, resulting in massive VEGF release and apoptosis of rMCs (83).

Oxidative stress is recognized as a vital mediator of DR, and the impairment of retinal cells is also triggered by the accumulation of ROS. Chen et al. (84) found that high glucose resulted in the excessive expression of oxidant species with a crucial role in senescence, which could be reversed by the antioxidant NAC. Additionally, lipid metabolism was related to the accumulation of hydrogen peroxide (H_2_O_2_) and subsequent senescence in ARPE-19. Activation of the PI3K/Akt/mTOR signaling pathway was responsible for the generation of intracellular lipids via modulation of fatty acid synthesis, which may in turn control senescence. Moreover, autophagy was enhanced in ARPE-19 exposed to HG for 48 h, and autophagy inhibition further deteriorated senescence with an increase in oxidant species. Prolonged HG exposure (14 days) significantly prohibited autophagy and stimulated apoptotic cells. Therefore, short-term exposure of hyperglycemia may induce autophagy to protect RPE from oxidative stress-induced damage, while long-term HG exposure inhibits autophagy and enhances apoptosis (84).

As glucose is a crucial metabolic component of the retina, DM patients must strictly sustain normoglycemia since either hyperglycemia or hypoglycemia will accelerate the development of DR. Low glucose disrupts the subtle equilibrium between autophagy and apoptosis. For example, Balmer et al. (85) found that retinal cell death is induced by acute hypoglycemia both *in vitro* in 661W photoreceptor cells exposed to low glucose and *in vivo* using an hyperinsulinemic/hypoglycemic clamp. In retinal cells, hypoglycemia-induced apoptosis was increased in parallel with autophagosome formation by the activation of the AMPK/RAPTOR/mTOR pathway. However, the autophagosome/lysosome fusion process was obstructed following decreased LAMP2a expression. Specific inhibition of autophagy promoted apoptosis and 661w cell death. Thus, cells resisted low nutrient condition-induced apoptosis by activating an autophagic process, which resulted in cell death. The autophagy dysfunction is related to 661W cells death caused by low glucose, and short-term regulation of autophagy could be envisioned to protect diabetic patients from secondary complications of the disease (85).

The findings of the above studies demonstrate cross talk in the retina between autophagy and apoptosis in the progression of DR. Enhanced autophagy could protect retinal cells under diabetic conditions by inhibiting apoptosis induced by DR stress.

### The Roles of Autophagy in Maintaining BRB

#### The Inhibition of Autophagy Induced by Hyperglycemia in Inner BRB

DR impairs sight via neuronal and vascular dysfunction in the retina. The inner BRB consists of ECs with tight junctions that are almost impermeable to protein transport. Alterations to autophagy in inner BRB have been studied (86). The levels of miR-204-5p and VEGF were significantly increased in retinal tissues of streptozocin (STZ)-induced diabetic rats compared to a control group. The ratio of LC3B-II/LC3B-I was significantly downregulated in the diabetes group compared with the control. *In vivo* in diabetic rats and *in vitro* in retinal endothelial cells isolated from diabetic rats, anti-miR-204-5p treatment significantly improved the expression level of LC3B-II/LC3B-I, and this change was suppressed in response to miR-204-5p mimic transfection. Thus, autophagy activation by miR-204-5p inhibition in retinal endothelial cells could mitigate DR (86).

High glucose and insulin (HGI) in human retinal endothelial cells leads to a marked increase in expression of migration-related genes, including matrix metalloproteinase-2 (MMP-2) and MMP-9. Angiogenesis-related genes, including hypoxia-inducible factor 1α (HIF-1α), VEGF, and insulin-like growth factor-1 (IGF-1) were also upregulated to the transcriptional and translational levels. However, no clear effect on the expression level of LC3 was observed. When Sirt3 was upregulated by lentivirus infection under HGI conditions, the increase of MMP-2, MMP-9, HIF-1α, VEGF, and IGF-1 were inhibited significantly. Meanwhile, LC3 mRNA, and LC3-II protein were enhanced. This study revealed that Sirt3 might ameliorate retinal neovascularization by modulating the expression of migration-, neovascularization- and autophagy-related factors (87).

#### The Protective Role of Autophagy in Outer BRB

RPE cells help to maintain fluid balance within the retina, by constituting the outer BRB and supporting photoreceptor function. ARPE-19 cells cultured under HG (25 mM) or hypoxia (1% oxygen) showed increased phosphorylation of the stress-activated kinases JNK and p38 MAPK. Both conditions also led to activated phosphorylation of the ER stress markers including PERK and eIF2a and elevation of the pro-apoptotic transcription factor CHOP, but inhibited autophagy as revealed by the downregulation of LC3II/I. Additionally, under these experimental conditions, ROS were induced and the integrity of tight junctions was impaired. Fenofibric acid (FA) had the opposite effect, protecting ARPE-19 cells against hyperglycemia- or hypoxia-caused harmful effects. FA may also increase insulin-like growth factor I receptor (IGF-IR)-mediated survival signaling in cells exposed to hyperglycemia and hypoxia, thereby inhibiting caspase-3 activation and downregulation of BclxL. Furthermore, FA upregulated LC3-II and thus increased autophagy flux. Therefore, FA elicited a dual protective effect on RPE by inhibition of stress-mediated signaling and induction of autophagy and survival pathways to improve the cell viability and sustain the integrity of BRB (88).

Bermúdez et al. (89) studied the inflammatory response in DR, with ARPE-19 and D407 cultured in lipopolysaccharide (LPS, 10 or 25 μg/ml). They explored the autophagic flux and its regulation by the phospholipases D (PLD) pathway in cells exposed to LPS. The LPS treatment triggered LC3B-II content and LC3B-positive punctate structures in both of the RPE cell lines. LC3B-positive punctate structures were elevated in cells in a control condition with PLD2 inhibitor pre-incubation, while LC3B was enhanced in cells exposed to LPS with PLD1 inhibitor. Moreover, 3-MA increased the loss in cell viability instigated by LPS. Therefore, although LPS treatment induced an inflammatory activation in RPE cells, it also promoted the activation of the autophagic process which in turn may elicit a protective effect on the cells via PLD pathway modulation (89).

## The Detrimental Role of Autophagy in Diabetic Retinopathy

### Interdependent Relationships Among Oxidative Stress, ER Stress, Apoptosis, and Autophagy in Deteriorating DR

Emerging evidence reveals that oxidative stress-induced autophagy and apoptosis of retinal cells are closely related to the pathogenesis of DR. It is well-known that diabetes will result in increased ROS, inflammation, and apoptotic cell death (90). ROS accumulation also enhances the activation of autophagy, which works to maintain cellular homeostasis (91). Baseline autophagy can prevent cells from oxidative stress by diminishing harmful intracellular materials, while excessive levels may lead to autophagic death of retinal cells. Cai et al. (92) explored the apoptosis and autophagy induced by oxidative stress in retinas of type 2 diabetic rats, induced by high fat and sugar intake and a single injection of STZ (40 mg/kg). They found that oxidative stress-related enzymes including NOX3 and SOD2 were enhanced, andapoptosis and LC3II/I were increased, along with the activation of phosphorylated ERK1/2 and phosphorylated AKT. The enhanced autophagy and apoptosis accelerated DR progression. Glucagon-like peptide-1 (GLP-1), an endogenous insulin tropic peptide, is delivered by L-cells via food ingestion and is considered a potential treatment for diabetes when applied at effective concentrations (93). In addition, GLP-1 may alleviate autophagy and apoptosis for the suppression of oxidative stress, likely through the GLP-1R-ERK1/2-HDAC6 signaling pathway. These findings revealed that the inhibition of autophagy and apoptosis by downregulating oxidative stress could mitigate DR.

TXNIP is recognized as a pro-inflammatory, pro-oxidative stress, and pro-apoptotic protein in diabetes, chronic hyperglycemia, and cellular stress. Devi et al. (94) found that HG increased TXNIP in retinal Müller cells obtained from STZ-induced diabetic rats. HG induced continuous upregulation of ROS generation, ER stress activation, ATP depletion, inflammation, and enhanced autophagy and apoptosis. These findings revealed that different mechanisms are induced by hyperglycemia, including NLRP3 inflammasomes, ER stress response, HIF-1α activation, autophagy/mitophagy, and apoptosis. TXNIP depletion inhibited ROS generation, recovered ATP level, and decreased autophagic LC3B in rMC1. The study confirmed that HG could induce TXNIP overexpression in Müller glia and evoke a process of cellular defense or survival mechanisms that ultimately resulted in oxidative stress, ER stress, inflammation, apoptosis, and autophagy, subsequently exacerbating the development of DR (94).

Dyslipoproteinaemia is a well-known risk factor for atherogenesis in people with and without diabetes. Extravasation of plasma components, including lipoproteins, is usually blocked by the inner and outer BRB. Fu et al. investigated the effects of extravasated and modified low density lipoprotein (LDL) on different types of retinal cell or tissue in DR (95–97). Oxidative and ER stress was induced when human retinal capillary pericytes (HRCP) were cultured with heavily oxidized glycated LDL (HOG-LDL), leading to apoptosis, autophagy, and mitochondrial dysfunction. In a diabetic mouse model with hyperlipidemia (compared with mouse models of either diabetes or hyperlipidemia), retinal ER stress was increased. Additionally, ER stress was upregulated in diabetic human retina and correlated with the severity of DR. These findings revealed that modified LDL caused oxidative stress and ER stress, leading to autophagy stimulation and pericyte loss in DR (95). In retinas from patients with or without DR, extravasated LDL was increased. Exposure of human RPE cells to HOG-LDL (50, 100, 200, and 300 μg/ml) caused cell apoptosis, ROS accumulation, ER stress, and decreased cell viability. While pretreatment with N-HDL had a protective influence, HOG-HDL was less effective. In DR conditions, extravascular modified LDL may worsen RPE impairment by oxidative stress, ER stress, apoptosis, and autophagy. Extravasation and modification of HDL may mitigate the harmful effects of extravasated modified LDL on the RPE in the development of DR (96). In cultured human Müller cells (MIO-M1), N-LDL had no effect, but HOG-LDL demonstrated significant toxicity, inhibiting cell viability and stimulating oxidative stress, apoptosis, autophagy, inflammation, expression of angiogenic factors, and glial cell activation Berberine may attenuate modified LDL-induced Müller cell injury by activating the AMPK pathway to inhibit autophagy, and is a potential target for the amelioration of DR (97).

To study histone modifications in the development of DR, Wang et al. (98) conducted experiments on the histone variants and autophagy in DR. In the retinas of type 1 diabetic rodents, enhanced levels of autophagy and histone HIST1H1C/H1.2 (an important variant of the linker histone H1) were observed. Upregulation of histone HIST1H1C increased HDAC1 and SIRT1, leading to elevated expression of ATG proteins and stimulation of autophagy in cultured rMC-1 and 293T cells. Histone HIST1H1C upregulation also induced cellular inflammation and injury, while downregulation reduced both the basal and HG-induced autophagy and prevented inflammation and cell damage. Verified by *in vivo* experiments, AAV-mediated histone HIST1H1C overexpression in the retinas resulted in enhanced autophagy, glial induction, inflammation, and neuron loss, similar to the pathological alterations in the early phase of DR. Conversely, downregulation of histone HIST1H1C by siRNA transfection in diabetic retinas markedly ameliorated the diabetes-induced autophagy, glial activation, inflammation, and neuron loss. Therefore, upregulated histone HIST1H1C promoted autophagy and impairments in the early stage of DR (98).

In rMC-1 cells treated with CoCl_2_ (300 μM) to induce chemical hypoxia, cell viability was inhibited by enhanced apoptosis, while activated phosphorylated AMPK and suppression of mTOR induced autophagy (99). However, lutein was found to improve Müller cell viability and promote cell survival in response to hypoxia by modulation of the intrinsic apoptotic pathway. Lutein treatment also inhibited autophagosome formation under hypoxic conditions, and may suppress autophagic flux even after pharmacological (rapamycin) stimulation of autophagy. Therefore, lutein may have a protective role, improving glial cell survival after hypoxic injury by inhibiting autophagy and apoptosis (99).

GLP-1, has been considered as a crucial intelligent hypoglycemic agent for its various biological effects, involving sufficient glycemic control, lipid metabolism, blood pressure reduction, weight loss, organ preservation, and significant neurotrophic action, by binding with the receptor, GLP-1R (100). Liraglutide, as a GLP-1 analog, effectively alleviated H_2_O_2_-induced cell viability reduction, mitochondrial morphological deterioration, and stimulation of autophagy, with elevated expression of LC3 II/I and Beclin-1, accompanied by p62 decline. However, rapamycin may induce autophagy and block these protective effects. Liraglutide also maintained mitochondrial protection and ameliorated H_2_O_2_-induced ROS overproduction, partially by enhancing mitochondrial generation and attenuating mitophagy. Thus, liraglutide could protect against H_2_O_2_-induced RGC-5 cell damage by downregulating autophagy and sustaining a balance between mitochondrial biogenesis and mitophagy (101).

Transactive response DNA binding protein 43 (TDP-43) belongs to the heterogeneous nuclear ribonucleoprotein family and is a well-recognized RNA-binding protein. It has been revealed that TDP-43 is associated with the pathogenesis of neuronal degenerative diseases (102). TDP-43 was found to be highly expressed in RGC-5 cells exposed to H_2_O_2_, and knockdown of TDP-43 significantly mitigated RGC-5 cell injury caused by H_2_O_2_. Additionally, repression of TDP-43 significantly inhibited both H_2_O_2_-induced oxidative stress and the production of intracellular ROS. Moreover, downregulation of TDP-43 decreased H_2_O_2_-triggered autophagy, with reduced expression of Beclin-1, p62, and LC3II/I. Suppression of TDP-43 eliminated the expression of histone deacetylase 6 (HDAC6), and HDAC6 also diminished the inhibitory effect of TDP-43 downregulation on H_2_O_2_-induced autophagy and apoptosis. Hence, the silencing of TDP-43 could protect RGC-5 cells against oxidative stress-mediated autophagy and apoptosis by inhibiting HDAC6 in DR (103).

### Autophagy Deteriorates the Neovascularization and Vascular Damage in DR

Angiogenesis is a physiological process included in neovascularization or growth of new vessels, and it is a dynamic component of embryological growth, tissue development, and wound healing in damaged tissues (104). However, inadequate vessel growth or disordered remodeling reportedly deteriorates diseases involving inflammation, cancers, and eye diseases such as DR (105). Thus, whether and how autophagy participates in the angiogenesis resulting from high glucose has been investigated. RF/6A cells were randomly separated into different groups (control, low glucose, HG 25 mM, and HG with 3-MA). Hyperglycemia significantly decreased cell viability and increased tube formation and cell migration, thus promoting angiogenesis. Meanwhile, ROS production and autophagy were found to be enhanced in the HG conditions, but pretreatment with 3-MA improved cell viability and reduced tube formation and cell migration, as well as decreasing ROS accumulation and autophagy. Therefore, inhibition of autophagy may help to mitigate the progression of DR by decreasing the formation of HG-induced retinal neovascularization (106).

Similarly, Li et al. (107) found that the exposure of RF/6A cells to HG (25 mM) inhibited cell proliferation, and promoted tube formation and cell migration, suggesting accelerated angiogenesis. Autophagy was induced in HG, revealed as increased LC3B fluorescent dots and enhanced level of LC3II/I, ATG5 proteins as well as downregulated p62. Adiponectin, which is an adipose-derived hormone with metabolic activity, has the potential to inhibit angiogenesis of endothelial cells (108). Treatment with adiponectin or 3-MA reverted the detrimental effects caused by hyperglycemia in RF/6A and downregulated autophagy by activating the expression of p-PI3K, p-AKT, and p-mTOR compared to the normoglycemic situation. Therefore, adiponectin could inhibit HG-induced angiogenesis of RF/6A cells, and ameliorate DR, by decreasing autophagy mediated by the PI3K/AKT/mTOR pathway (107).

High expression of miR-1273g-3p has been found in RPE cells extracted from retinas of diabetic rats (109). MiR-1273g-3p mimic enhanced the expression of DR-related MMP-2, MMP-9, and TNF-α proteins, and autophagy-related LC3II/I, cathepsin B, and cathepsin L factors, while miR-1273g-3p inhibitor could downregulate the levels of these factors. Thus, miR-1273g-3p is involved in the progression of DR by modulating the autophagy-lysosome pathway (109).

In a study on the morphological, electrophysiological and biochemical changes in STZ-induced diabetic mouse retinas, enhanced LC3 immunostaining at the outer plexiform layer (OPL) and elevated levels of Beclin-1 and ATG5 were observed, but significant apoptotic signs were not detected. Eight weeks after diabetes induction, rod but not cone photoreceptors were lost, and thickness of the inner and outer synaptic layers was reduced. Consistent with these findings, rhodopsin expression was downregulated and the scotopic electroretinogram (ERG) was reduced, while cone opsin expression and photopic ERG response were unchanged. Reduction of the scotopic ERG preceded morphological alterations and any detectable sign of vascular change, suggesting that loss of rod function indicates the beginning of STZ-induced DR in mouse. The autophagic pathway was enrolled in initial impairment of the rod pathway. The pathogenic pathways causing cell death proceeded with the activated dysregulation of autophagy prior to vascular damage but without strong modulation of apoptosis in DR (110).

## Overview of Dysregulated Autophagy in Diabetic Nephropathy

Diabetic nephropathy (DN) is another serious complication of diabetes mellitus, which is a leading cause of end-stage kidney disease (111). Accompanying with the renal injury, diabetic patients often suffer from kinds of complications, including retinopathy, neuropathy, and cardiovascular diseases (112). Additionally, there are common stress and signaling pathways inducing dysfunctional autophagy between DR and DN. Generally speaking, while kidney cells are suffered from stress conditions, including hyperglycemia, hypoxia, ER stress, oxidative stress, and genotoxic damage, autophagy is aroused and plays an essential role for cell survival (113). Under the condition of nutrient/energy excess, autophagy is inhibited (114). Although the down-regulation is protective in short term, defective autophagy may eventually subscribe to the accumulation of cellular damage, leading to the progression of metabolic kidney dysfunctions.

Emerging evidence has revealed that autophagy is impaired in tubular and glomerular cells under diabetic conditions with both type 1 and 2 DM. Autophagy was inhibited in proximal tubules of STZ-induced early diabetic rats, related to tubular hypertrophy (115). Similar observations were also found in podocytes from STZ-induced diabetic mice, as indicated by p62/SQSTM1 accumulation (116). Additionally, an increase of p62 in proximal tubule epithelial cells (PTECs) in kidney biopsy from type 2 DM patients was further detected, revealing that human diabetic kidneys are also insufficient in autophagy activity (117). Despite the capacity for autophagy is suppressed in diabetic kidneys, the demand for protective autophagy is dramatically enhanced owing to a high exposure to cellular stresses induced by diabetes. This contradictory autophagic reaction may accelerate the development of renal damage in DN.

Further exploring the regulation and roles of autophagy in the pathogenesis of DN, nutrient-sensing pathways and intracellular stress signaling were focused on. The well-recognized nutrient-sensing pathways involve the mTOR, AMPK, and SIRT. Overexpression of mTORC1 is observed in animals and patients with diabetic kidney disease, with the inhibition of autophagy (118, 119). By contrast, downregulation of mTORC1 by rapamycin or knocking down has renoprotective effects against DN, suppressing pro-inflammatory and profibrotic cytokines, inhibiting mesangial expansion, glomerulosclerosis, proteinuria, and renal hypertrophy (120, 121). Taken together, the suppressed autophagy induced by hyperactivation of mTOR signaling pathway, could deteriorate the pathogenesis of DN. While, the activity of AMPK in the kidney is suppressed in experimental type 1 and 2 diabetic models (122, 123). Stimulation of AMPK could attenuate the development of DN by enhancing autophagy, reducing oxidative stress, prohibiting high glucose-induced matrix protein synthesis, and reversing endothelial dysfunction (124, 125). Similarly, the expression of SIRT1 is decreased in renal cells in human and animal models of DN, and overexpression of SIRT1 protects the kidney from diabetic injury with the activation of autophagy (126). Together, these findings suggest that under diabetic conditions, stimulation of mTORC1 and/or decreased AMPK and SIRT1 inhibit autophagy and thus accelerate the progression of the renal disease. However, as a compensatory reaction, autophagy is activated by the stresses signaling including oxidative stress, ER stress and hypoxia to sustain cell homeostasis. Once this adaptive struggle fails, impaired organelles, such as ER and mitochondria, may accumulate in cells and deteriorate DN (127). In early stage of diabetes, autophagy can be induced by ROS generation, which is aroused by hyperglycemia, damaged mitochondria, advanced glycation and et. al. ROS activates autophagy through activating PERK-eIF2α (128) and JNK1 (129) pathways. Therefore, oxidative stress can promote autophagy to clear damaged mitochondria (mitophagy) (130). This autophagy-mediated mitochondrial control and subsequent decreasing of ROS should be crucial to protect kidney from diabetic injuries. ER stress can be enhanced by hyperglycemia, and it induces autophagy through the unfolded protein response (UPR) with mediating the transcriptional activation of LC3 and ATG5 (131, 132), while the defective autophagy reversely leads to prolonged ER stress and kidney injury under the diabetic condition. Inhibition of ER stress exerted the renoprotective effects by reactivation of autophagy in DN conditions. Moreover, hypoxia can stimulate autophagy through both HIF-dependent and -independent mechanisms (133, 134). HIF1 is induced by hypoxia and activates autophagy through transcriptional increasing of BNIP3 and BNIP3 like (BNIP3L/NIX) to release BECN1 from its inhibitory interaction with BCL2. Severe hypoxia (<0.01% oxygen) can also enhance ER stress to stimulate autophagy through the UPR pathway. In addition, by activating AMPK pathway, hypoxia can activate TSC1/2 and thereby reduce mTORC1 activity to induce autophagy. Based on the above researches, regulation of autophagy coordinated by these signaling pathways in diabetic kidneys would protect against diabetic renal injury in DN. Referring to the common regulatory mechanisms of autophagy in DR and DN, agents targeted for treating DN may hint the potential therapeutic application for DR.

## Conclusion and Perspectives

DR is a serious microvascular complication of DM, with underlying pathogenic mechanisms derived from multiple risk factors. Hyperglycemia at an early stage and hypoxia at later stages of DR are two of the main aggressors related to retinal pathologic alterations, including induction of ischemia, oxidative stress, ER stress, inflammation, and altered growth factors. Autophagy is a catabolic pathway responsible for the degradation and recycling of cellular components under normal conditions, and is thought to be part of the fate of stressed cells. Emerging evidence has revealed that autophagic signaling, with concomitant modulation and dysregulation, tends to occur in the pathophysiological process of DR. Whether high glucose and hypoxia in DR cause an increase in autophagic flux or suppress autophagy has not been established unambiguously.

**Figure 2:**
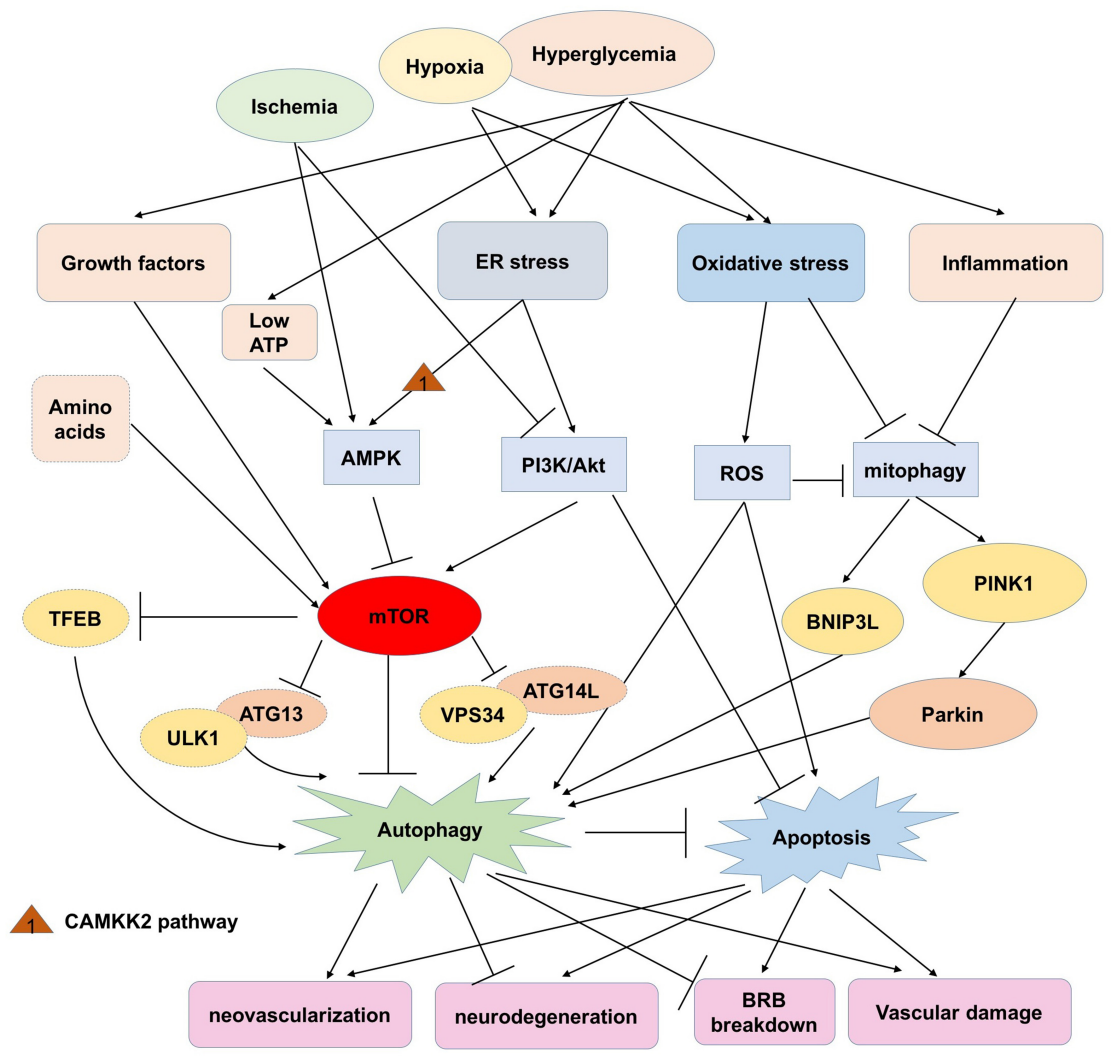
The potential roles and related pathways of autophagy in DR.

This review highlights the dual roles of autophagy in sustaining retinal and cellular functions, (Table 1) but also deteriorating retinal dysfunction in the progression of DR (Table 2). Generally, hyperglycemia or hypoxia cause excessive oxidative stress, ER stress, and inflammation, which then activate the AMPK pathway, modulate PI3K/Akt signaling and subsequently regulate mTOR. With the accumulation of ROS or inflammatory factors, mitophagy is activated abnormally and unable to eliminate the damaged mitochondria in response to intra- and extracellular stress. Autophagic flux is induced in the early stage of DR by the inhibition of mTOR, and exerts protective roles in neuron injury, anti-apoptosis, sustaining the BRB and inhibiting the inflammatory response. However, overexpression of autophagy with lysosome dysfunction may aggravate cell death, retinal neovascularization, and vascular damage (Figure 2). Considering that the effects of individual variations and environmental factors might play vital roles in different kinds of retinal cells mimicking DR, the role of autophagy in DR needs more studies to be better understood.

**Table 1:** The mechanisms involved in protective roles of autophagy in DR.

**Retinal cell or tissue**	**Circumstance**	**Autophagy-related markers**	**Related pathways**	**Role of autophagy**	**References**
RGC	Retinal ischemia mouse model	LC3II ↗↘ p62/SQSTM-1 ↑ ATG proteins ↓	PI3K/Akt/mTOR ↓ AMPK ↑	Neuroprotection	(58)
*Ex vivo* mouse retinal explants	HG (75 mM)	LC3II ↓ p62 ↑	mTOR ↑ apoptosis ↑	Neuroprotection	(59)
RGC	STZ-induced diabetic rats (60 mg/kg)	LC3II/I ↑ Beclin-1 ↑	mTOR ↓ AMPK ↑	Neuroprotection	(60)
rMC-1 retina of db/db mice	HG (60 mM)	LC3II/I ↑ p62/SQSTM-1 ↓	PINK1 ↑ Parkin ↑	Mitophagy	(68)
Primary mouse Müller cells/human MIO-M1 cells	HG (30.5 mM) Diabetic mouse retina	LC3II/I ↑ p62/SQSTM-1 ↓	Pink1 ↑ Park2 ↑	Mitophagy	(69)
ARPE-19	HG (50 mM)	LC3II/I ↓ p62/SQSTM-1 ↑	ROS ↑ PINK1/Parkin ↓	Mitophagy Anti-apoptosis	(70)
ARPE-19	HG (30, 50, and 70 mM)	LC3II ↑ LC3I ↓	ROS ↑ PINK1/Parkin ↑ BNIP3L ↑	Mitophagy	(71)
ARPE-19	HG (30 mM)	LC3II/I ↑	ROS ↑ NLRP3 inflammasome ↑	Mitophagy inflammasome inhibition	(73)
Primary rat Müller cell	HG (40 mM)	LC3II/I ↓ p62/SQSTM-1 ↑	mTOR ↑	Anti-apoptosis	(82)
rMC-1 STZ-induced Wistar Kyoto rats (60 mg/kg)	HG (25 mM)	LC3II/I ↑ p62/SQSTM-1 ↑ Beclin-1 ↑	ER stress ↑	Anti-apoptosis	(83)
ARPE-19	HG (25 mM)	LC3II/I ↑↓ p62/SQSTM-1 ↓↑	ROS ↑ PI3K/Akt/mTOR ↑	Anti-apoptosis	(84)
661W photoreceptor cells	LG (1 mM) HG (25 mM)	LC3II/I ↑↓ p62/SQSTM-1 ↑	AMPK ↑ RAPTOR/mTOR ↑	Anti-apoptosis	(85)
Rat retinal endothelial cells (rREC)	STZ-induced diabetic rats (40 mg/kg)	LC3II/I ↓	/	Protect inner BRB	(86)
HREC	HGI (HG 25 mM + insulin 100 nM)	LC3II/I (-)	Sirt3 ↓ MMP-2/MMP-9 ↑ VEGF/HIF-1α/ IGF-1 ↑	Protect inner BRB	(87)
ARPE-19	HG (25 mM) Hypoxia	LC3II/I ↓	ROS ↑ JNK/p38 MAPK ↑ ER stress ↑	Anti-apoptosis Containing outer BRB	(88)
ARPE-19 D407	LPS (10/25 μg/ml)	LC3II/I ↑ p62/SQSTM-1 ↑	ROS ↑	Anti-inflammation	(89)

*Blue arrows indicate increasing, and red arrows indicate decreasing*.

**Table 2:** The detrimental effects of autophagy on DR.

**Retinal cell or tissue**	**Circumstance**	**Autophagy-related markers**	**Related pathways**	**Role of autophagy**	**References**
Retina tissues	Diabetic rats (high fat and sugar; STZ 40 mg/kg)	LC3II/I ↑	SOD2/NOX3 ↑ Akt/ERK1/2 ↑	Oxidative stress Apoptosis	(92)
rMC-1 Retina tissues	HG (25 mM) STZ-induced diabetic rats (65 mg/kg)	LC3II/I ↑ Beclin 1 ↑	ROS ↑ ER stress ↑	Oxidative stress Inflammation Apoptosis	(94)
Human retinal capillary pericytes (HRCP)	HOG-LDL; A mouse model of diabetes and hyperlipidaemia	LC3II/I ↑	ROS ↑ ER stress ↑	Oxidative stress Apoptosis	(95)
Human RPE (hTERT-RPE) cells	HOG-LDL Human retinas (NC/DM)	LC3II/I ↑ Beclin 1 ↑ ATG 5 ↑	ROS ↑ ER stress ↑	Oxidative stress Apoptosis	(96)
Human Müller cells (MIO-M1)	HOG-LDL	LC3II/I ↑ Beclin 1 ↑ ATG 5 ↑	ROS ↑ ER stress ↑	Oxidative stress Apoptosis Inflammation	(97)
rMC-1 293T cells retinas	HG (25 mM) STZ-induced diabetic rats Ins2^+/−^ mice	LC3II/I ↑ BECN1 ↑ ATG12-5 ↑	Histone-HIST1H1C ↑ HDAC ↑	Inflammation apoptosis	(98)
rMC-1	Hypoxia CoCl_2_ 300 μM	LC3II/I ↑	pAMPK ↑ mTOR ↑	Apoptosis	(99)
RGC-5 cells	H_2_O_2_ (20 μM)	LC3II/I ↑ Beclin 1 ↑ p62 ↓	ROS ↑ Parkin ↑	Mitophagy	(101)
RGC-5 cells	H_2_O_2_ (200 μM)	LC3II/I ↑ Beclin 1 ↑ p62 ↓	ROS ↑ HDAC6 ↑	Oxidative stress apoptosis	(103)
RF/6A	HG (25 mM)	LC3II/I ↑ p62 ↓ ATG 7 ↑	ROS ↑	Neovascularization	(106)
RF/6A	HG (25 mM)	LC3II/I ↑ p62 ↓ ATG 5 ↑	PI3K ↓ Akt/mTOR ↓	Neovascularization	(107)
Primary rat RPE cells	Diabetic rats (high fat; STZ 20 mg/kg)	LC3II/I ↑	MMP-2 ↑ MMP-9 ↑ TNF-α ↑	Vascular damage	(109)
Retinas	STZ-induced diabetic mice (150 mg/kg)	LC3II/I ↑ Beclin 1 ↑ ATG 5 ↑	/	Rod impairment	(110)

*Blue arrows indicate increasing, and red arrows indicate decreasing*.

More researches are needed to resolve the controversy around the influence of autophagy in the progression of DR, by exploring the relevant upstream molecular pathways modulating autophagy and the downstream signals determining the impact of autophagy in DR on the essential retinal cells. Research of this kind will help to understand the protective role of autophagy in DR, and possibly identify novel targets for early intervention and protection of retinal cells in various retinopathies. It can be anticipated that by developing compounds which selectively induce or target autophagy may have exciting therapeutic perspective in the different stages of DR. Although several crucial questions need to be further addressed before these innovative agents can be applied in a clinical trial, the research field of autophagy in DR is advancing quickly and clinically connected applications on these topics may be foreseen soon. Detailed characterization and roles of autophagy in different stages of DR in combination with the efficient clinical interference of autophagy regulation proposes exciting new avenues for the development of therapeutic strategies in DR.

## Author Contributions

QG, TQ, and XX conceived and designed the review. QG, HW, and PY collected the literature and performed the analysis. QG and HW wrote the manuscript. TQ and XX critically revised the manuscript and provided the funding. All authors have revised the manuscript for important intellectual content and approved the final version to be published.

## Conflict of Interest

The authors declare that the research was conducted in the absence of any commercial or financial relationships that could be construed as a potential conflict of interest.
